# Nigeria’s efforts to strengthen laboratory diagnostics – Why access to reliable and affordable diagnostics is key to building resilient laboratory systems

**DOI:** 10.4102/ajlm.v9i2.1019

**Published:** 2020-08-26

**Authors:** Dhamari Naidoo, Chikwe Ihekweazu

**Affiliations:** 1 WHO Health Emergency Program, Infectious Hazard Management, World Health Organisation, Abuja, Nigeria; 2 Nigeria Centre for Disease Control, Abuja, Nigeria

## Introduction

Since 2016, the Nigeria Centre for Disease Control (NCDC) has been strengthening its technical capacities as the country’s national public health institute.^1^ One key area receiving considerable attention has been the strengthening of laboratory diagnostic and networking capabilities for diseases of public health importance. Previous investments in strengthening and financing laboratory capacities have focused almost entirely on single disease programmes. In just over two years, the NCDC has made progress in changing this situation with the operationalisation of a new National Reference Laboratory. The National Reference Laboratory has the diagnostic capacity for national priority diseases, including yellow fever (YF), Lassa fever, monkeypox, cerebrospinal meningitis, cholera, influenza and other enteric pathogens. In addition, a specimen referral system has been established to cover the large geographic expanse of the country and a national laboratory network supporting 41 facilities. The national laboratory network integrates vertical disease networks for the standardisation of testing algorithms and improvement of coordination and functionality.^2^

In 2017 alone, the NCDC responded to widespread outbreaks of cerebrospinal meningitis, monkeypox, YF, Lassa fever and cholera.^1^ With several public health challenges, including increasing disease outbreaks and emerging antimicrobial resistance,^3^ efficient coordination and integration of surveillance and laboratory systems is of critical importance. Improving access to diagnostics at the country level is a fundamental component of building laboratory systems critical for disease surveillance and timely outbreak detection and containment, as well as improved patient management.^4^

Advocacy on the importance of diagnostic development for outbreak preparedness is occurring through global strategies such as the World Health Organization’s (WHO) Research and Development Blueprint and Eliminating Yellow Fever Epidemics strategy.^5,6^ However, challenges still remain in accessing diagnostic tests that are reliable, available through local production or procurement, and easily implementable and scalable during emergencies.^7^ This is becoming more apparent as countries struggle to leverage laboratory capacity from established vertical disease programmes, such as polio, HIV and tuberculosis, for emerging public health threats. The limited global investments to increase access to diagnostics across a wider range of diseases of public health concern, rather than individual diseases, will continue to limit the capacity of African countries to develop resilient and disease-wide public health systems.^4^ This report summarises the challenges and early successes of laboratory integration across two disease networks in Nigeria.

## Challenges in expanding and enhancing yellow fever laboratory diagnostics

**FIGURE 1:**
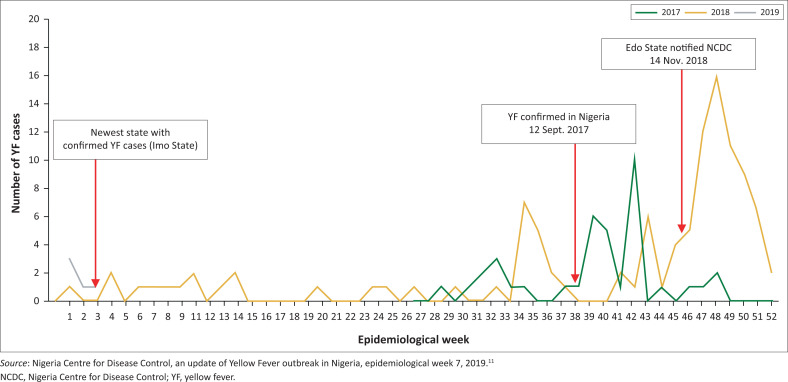
Weekly trends of yellow fever outbreak in Nigeria from 2017 to 2019.

Since 2016, the re-emergence of YF has caused outbreaks in Angola, Brazil, Chad, the Democratic Republic of Congo, Ghana, Guinea and Uganda.^8^ This prompted the WHO to launch the Eliminating Yellow Fever Epidemics strategy to reduce the risk of YF in high-risk countries through strengthening outbreak detection, response and prevention.^9^ In 2017, YF re-emerged in Nigeria, 17 years since the last reported case.^10^ Localised outbreaks continue to be reported and, as of March 2019, Nigeria had seen approximately 4100 suspect cases, of which 139 were confirmed in 17 states throughout the country^11^ (Figure 1).

**FIGURE 2:**
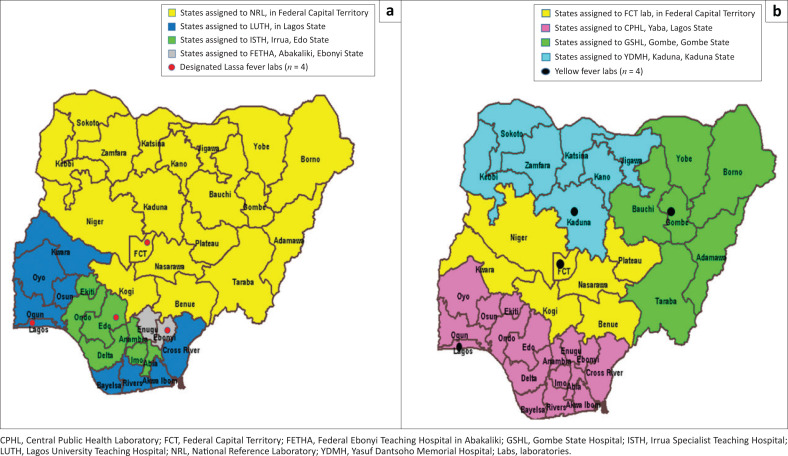
Geographic location and specimen referral catchment areas for the Lassa fever and yellow fever laboratories. (a) Lassa fever laboratories, (b) Yellow fever laboratories.

The Nigerian YF laboratory network, established in 2006, consists of four subnational laboratories located in Lagos, Kaduna, Abuja and Gombe (Figure 2), with capacity to implement imunoglobulin M (IgM) detection, the standardised African YF laboratory diagnostic algorithm. The national laboratories receive reagents for laboratory-developed, enzyme-linked immunosorbent assays through two sources: the Institut Pasteur of Dakar and the United States Centres for Disease Control and Prevention.^12^ Due to operational differences between the laboratory-developed and commercial ELISA protocols, implementation is challenging and requires continuous training for the national staff. Following recommended guidelines, presumptive positive samples from any of the four national laboratories have to be shipped to the WHO’s regional reference laboratory at the Institut Pasteur of Dakar in Senegal, where additional testing is conducted. This is done to exclude vaccine-induced imunoglobulin M antibody positivity and cross-reactivity because of infections with other flaviviruses – a limitation of serological testing.^8^

The response to the ongoing outbreak has been challenged by limited access to reagents. Between 2016 and August 2017, no yellow fever testing occurred in Nigeria because of a lack of reagents.^7^ When the outbreak was first detected in Kwara State, Nigeria, in August 2017, the same month that reagents became available, it took one month to confirm the laboratory result at the regional reference laboratory. The index case reported the onset of symptoms on 16 August 2017 and the final laboratory confirmation was only received on 12 September 2017.^9^ Delayed outbreak detection and reporting of cases has an impact on the approval of vaccine requests from the International Coordination Group on Vaccine Provision^13^ and planning for reactive vaccination campaigns for outbreak control.

The importance of rapid confirmation was recently highlighted during the widespread YF outbreak in Edo State, Nigeria, in 2018. Clusters of cases were reported from four local government areas on 14 November 2018. On 21 November 2018, the NCDC was informed of nine patient samples that had tested positive for YF by quantitative reverse transcription polymerase chain reaction (qRT-PCR) at the African Center of Excellence for Genomics of Infectious Disease, a non-YF-network laboratory. These YF cases were found in patients initially referred to the Irrua Specialist Teaching Hospital with suspected Lassa fever. After Lassa fever tests were negative, the patients were tested for YF following continued clinical presentation with typical viral haemorrhagic signs and symptoms.^14^ Immediately after receiving the report of positive results, the samples were sent to the Institut Pasteur of Dakar on 29 November 2018 for re-confirmation. The first set of results YF confirming both imunoglobulin M and qRT-PCR results were received from the Institut Pasteur of Dakar on 7 December 2018. The plaque reduction neutralisation test results were shared on 31 December 2018. However, the outbreak was declared on 24 November 2018, based on the qRT-PCR results.

Declaring the outbreak using the molecular results allowed for the immediate deployment of rapid response teams and the initiation of a request for vaccines from the International Coordination Group on Vaccine Provision. However, the challenge faced by national authorities was two fold – accepting test results carried out by non-WHO-recognised YF-network laboratory; and by qRT-PCR, a method not routinely used for confirmation at the national level. Molecular testing has not been effectively operationalised in the African region.^4,15^ In response, the national YF testing algorithm was updated to include molecular testing, and the national Lassa fever laboratories were registered in a YF qRT-PCR quality-assurance programme, a critical step required to demonstrate technical capacity to detect YF virus infection.

Overall, two critical bottlenecks hamper the strengthening of the national YF laboratory network. These are limited access to clinically validated or regulatory approved molecular and serologic tests either through the WHO network or through commercial manufacturers; and the limited capacity to perform in-country confirmatory diagnosis by plaque reduction neutralisation test and differential diagnosis for flaviviruses.^8^ The Eliminating Yellow Fever Epidemics strategy is currently addressing the shortfalls in diagnostics through updating diagnostic algorithms, working with industries and partners to fast-track diagnostic development and evaluations, and creating an international procurement and supply chain. To operationalise these changes at the country level, increased support and funding is required for training, procurement of diagnostic tests for YF and other flaviviruses, and access to quality control materials for the development of national external quality assessment activities.

## Building the Lassa fever laboratory network during an epidemic

In 2018, Nigeria recorded an unusually active epidemic season of Lassa fever. A total of 3498 suspect cases were reported, of which 633 were laboratory confirmed. Several factors, such as increased disease awareness, improved surveillance and laboratory capacity, were thought to have contributed to the increased number of confirmed cases.^3^

Before establishing national diagnostic capacity, testing was performed outside Nigeria, in Kenema, Sierra Leone. Between 2005 and 2012, diagnostic capacity was established at the Lagos University Teaching Hospital and the Irrua Specialist Teaching Hospital. The Irrua Specialist Teaching Hospital accounted for over 90% of the testing workload, as it provided diagnostic support for an endemic region in Nigeria.^16^ The 2018 epidemic season marked the beginning of a collaboration with the international community under the framework of the WHO’s Research and Development Blueprint to improve Lassa fever detection, treatment and prevention.^17^

One of the biggest gains made in 2018 was the rapid expansion of national diagnostic capacity, with the establishment of two additional testing facilities at the NCDC National Reference Laboratory in Abuja and the Virology Laboratory at the Federal Teaching Hospital in Abakaliki, Ebonyi state (Figure 2). The expansion was attributable to the availability of funding during the outbreak from donors and the WHO through the Federal government to the NCDC. This enabled the procurement of equipment and reagents, the building of infrastructure and increased political commitment from the Federal and State Governments of Nigeria. A key factor in the early success of the network was the adoption of a standardised testing algorithm based on a 2-gene target testing strategy using qRT-PCR, with a combination of both commercial- (RealStar^®^ Lassa fever RT PCR kit version 1.0, Altona Diagnostics, Hamburg, Germany) and laboratory-developed tests being used to detect all known lineages of Lassa fever virus.^16^

Published diagnostic literatures showed limited availability of commercial assays for Lassa fever diagnosis; none was WHO approved.^17^ However, through the expedited WHO Research and Development pathway, a new kit-based format of the laboratory-developed test was evaluated in Nigeria and submitted for review under the WHO’s Expert Review Panel for Diagnostics at the end of 2018. Because of comparable analytical data between the laboratory-developed and commercial kits, improved usability and an easier procurement process, the new kit format (RealStar^®^ Lassa fever RT PCR kit version 2.0, Altona Diagnostics, Hamburg, Germany) was adopted within the laboratory network despite the need to perform the assay as two singleplex tests.

Despite the remaining challenges, such as difficulties in importing large quantities of diagnostic kits considered as dual-use by German and European Union legislation, and sustainable funding to maintain national testing capacity at the current levels, the collaborations with technical partners such as the Bernhard Nocht Institute of Tropical Medicine, the Foundation for Innovative New Diagnostics and the WHO under the WHO Research and Development Blueprint initiative, proved to be instrumental in capacity building and development of national priorities for operational research. This multi-partner collaboration has delivered tangible benefits to the country by improving diagnostic preparedness for Lassa fever and demonstrating that investments made during outbreaks improve health systems.

## Access to diagnostics and laboratory systems integration

As the NCDC continues its journey to deliver on its mandate, stronger integration of surveillance and laboratory systems becomes critical for outbreak detection and response. Laboratory network integration will facilitate integrated electronic reporting systems, improve the use of diagnostic testing to guide patient care across all levels of the health system, and allow for better financial planning to allocate resources required to maintain operational activities in the face of dwindling external funding.

To improve Nigeria’s YF and Lassa fever outbreak detection and response, there is the need to integrate both systems networks into an efficient and effective laboratory surveillance system. To achieve this, YF would need to be included as part of a panel of recommended tests for a differential diagnosis on patients who test negative for Lassa fever. To operationalise this differential testing, access to commercial YF molecular assays with clinical validation data is required.^8^ Further product development is needed for safe and reliable Lassa fever point-of-care testing to improve access to diagnostic testing for patients in all hospital facilities across Nigeria.

The experience of Nigeria has shown that restrictive access to diagnostic tests as a result of unequal global investment in diagnostic development hinders the building of resilient and integrated laboratory systems. Stronger leadership is required from the WHO to address these challenges. Greater progress will be made in building laboratory systems at the country level when the framework is built on (1) multi-partner engagement with joint leadership and collaboration with the ministries of health and national public health institutes of affected countries, (2) international and domestic funding that ensures the sustainability of diagnostic testing to promote continuous product development and (3) a revised African regional laboratory strategy that promotes diagnostic stewardship and the uptake of new diagnostics for multi-pathogen testing to reduce vertical disease structures. This is not only important for Nigeria but for the entire continent.
